# The Functions of the Pneumogastric Nerves

**Published:** 1855-02

**Authors:** 


The Functions of the Pneumogastric Nerves. By Dr. Sequard. —I wish to take date of the discovery of two important facts relative to the physiology of the pneumogastric nerves.

I had already found and published that these nerves are the motor nerves of the vessels of the heart; I have since found that they are also the principal (if not the only) motor nerves of the small vessels of the lungs. This view is grounded on the following facts:

1st. The section of the pneumogastric nerves, in the cervical region, is followed by the dilatation (or paralysis) of the small vessels of the lungs, as well as it is by the dilatation of the vessels of the heart.

2d. The galvanisation of these nerves in the cervical region, after they have been carefully separated from the sympathetic, produces the contraction of the vessels of the lungs, as well as that of the vessels of the heart.

I will prove elsewhere that the dilatation of both the vessels of the lungs and those of the heart, which follows the section of the pneumogastric nerves, is the first and principal cause of the death which is the constant result of this operation.

---

On the Collodion Treatment of Orchitis. By M. Ricord.—M. Ricord tried the collodion treatment, as proposed by Bonnafont, in thirty-eight cases of blenorrhagic epididymitis; and the following are the conclusions at which he has arrived:—

1st, Elastic collodian causes less suffering than ordinary collodian, but more than the other modes of treatment.

2d, It is not so efficacious a therapeutical agent as was imagined.

3d, It does not allay the pain, nor effect a cure more promptly than other modes of treatment.

4th, Its action is principally manifested on engorgement of the subscrotal cellular tissue, and on inflammation of the scrotum itself.

5th, It is only a feeble mode of exerting compression.

6th, If collodion only acts through the medium of the cold resulting from the evaporation of the ether, the application of simple ether, or of cold to the scrotum, would answer equally well.

7th, It is not rational to believe that collodion can cure orchitis and epididymitis, simply by protecting the diseased parts from the action of the air.—*L'Union Mèdicale.*

